# Breastfeeding patterns and its determinants among mothers living with Human Immuno-deficiency Virus -1 in four African countries participating in the ANRS 12174 trial

**DOI:** 10.1186/s13006-017-0112-2

**Published:** 2017-05-02

**Authors:** Eric N. Somé, Ingunn M. S. Engebretsen, Nicolas Nagot, Nicolas Meda, Carl Lombard, Roselyne Vallo, Marianne Peries, Chipepo Kankasa, James K. Tumwine, G. Justus Hofmeyr, Mandisa Singata, Kim Harper, Philippe Van De Perre, Thorkild Tylleskar, for the ANRS 12174 Trial Group

**Affiliations:** 1 Centre for International Health, University of Bergen, Bergen, Norway; 2 National Health Research Institute, Centre National pour la Recherche Scientifique et Technologique, 10 BP 250 Ouagadougou, Burkina Faso; 3 INSERM UMR 1058, Pathogenesis and control of chronic infections, Montpellier, France; 4 Université de Montpellier, Montpellier, France; 5 Centre Hospitalier Universitaire, Montpellier, France; 6 Faculty of Health Sciences, Centre de Recherche International en Santé (CRIS), University of Ouagadougou, Ouagadougou, Burkina Faso; 7 South African Medical Research Council, Biostatistics Unit, Cape Town, South Africa; 8 Department of Paediatrics and Child Health, University of Zambia, School of Medicine, University Teaching Hospital, Lusaka, Zambia; 9 Department of Paediatrics and Child Health, College of Health Sciences, Makerere University, School of Medicine, Kampala, Uganda; 10 Effective Care Research Unit, University of Fort Hare, Eastern Cape, South Africa

**Keywords:** HIV infection, Exclusive breastfeeding, Vertical transmission, Prevention, Sub Saharan Africa, Risk factors, Cohort study

## Abstract

**Background:**

HIV-1 transmission rates have been reduced over the last decade, an estimated 2 million new infections per year arise, including 220,000 paediatric cases. The main post-natal HIV exposure is through breastfeeding, where both its duration and modality (exclusive or not) are associated with postnatal transmission. The ANRS 12174 trial compared HIV-1 postnatal transmission of 2 prophylaxis drugs for infants during lactation (lamivudine and lopinavir-ritonavir). Our objective has been to examine the feeding practices and the determinants of exclusive/ predominant (EPBF) or any breastfeeding among the participants of this trial in Burkina Faso, South Africa, Uganda and Zambia.

**Methods:**

Mothers infected with HIV-1 and their uninfected offspring were followed from day 7 after birth for 50 weeks, keeping monthly records of their feeding patterns. Feeding was classified into 3 categories: 1) exclusive breastfeeding during the first six months, only breast-milk being given to infant for 6 months, 2) predominant breastfeeding, breast-milk with liquid-based items being given, and 3) mixed feeding, other non-breast milk or solid food being given in addition to breast milk with or without liquid-based items. The categories were merged into 2 groups: EPBF applying to infants aged <6 months and mixed feeding applying to infants of any age. The feeding patterns have been given as Kaplan-Meier curves. A flexible parametric multiple regression model was used to identify the determinants of the mothers’ feeding behaviour.

**Results:**

A total of 1,225 mother-infant pairs provided feeding data from Burkina Faso (*N* = 204), South Africa (*N* = 213), Uganda (*N* = 274) and Zambia (*N* = 534) between November 2009 and March 2013. The mean maternal age was 27.4 years and the mean BMI was 24.5. 57.7 and 93.9% of mothers initiated breastfeeding within the first hour and first day, respectively. Overall, the median durations of any form of breastfeeding and EPBF were 40.6, and 20.9 weeks, respectively. Babies randomized to the lopinavir/ritonavir group in South Africa tended to do less EPBF than those in the lamivudine group.

Overall the group of mothers aged between 25 and 30 years, those married, employed or multiparous tended to stop early EPBF. Mothers living in Uganda or Zambia, those aged between 25 -30 years, better educated (at least secondary school level), employed or having undergone C-section stopped any breastfeeding early.

**Conclusions:**

There is a need to improve breastfeeding and complementary feeding practices of children, particularly those exposed to HIV and anti-retrovirals, taking into account context and socio-demographic factors.

**Trial registration:**

Clinical trial registration: NCT00640263.

**Electronic supplementary material:**

The online version of this article (doi:10.1186/s13006-017-0112-2) contains supplementary material, which is available to authorized users.

## Background

Worldwide, there are 2.6 million children <15 years old living with the Human Immuno-deficiency Virus (HIV). Mother-to-child transmission of HIV-1 (MTCT) through pregnancy, childbirth and breastfeeding are the main routes of transmission according to estimates made in 2015 through the United Nations programme on HIV/AIDS (UNAIDS) [1]. Even if transmission rates have been reduced over the last decade, an estimated 2 million new HIV-1 infections occur per year including 220,000 paediatric cases [1]. Improved prevention of mother-to-child transmission of HIV-1 (PMTCT) strategies and programme implementation strengthening are therefore needed. Postnatal HIV-1 exposure can be avoided by replacement feeding, but this has been detrimental in settings with a high child mortality [2–4]. Non-breastfed children will also be deprived of the many known benefits of breastfeeding - better survival rates, and immunological and nutritional status [5–9]. Developmental [10] benefits include bonding of the mother-infant dyad, natural spacing of pregnancies [11] and cultural acceptability [12]. Mixed feeding seems to be the most risky option during the first 6 months of life regarding HIV transmission for infants born to mothers living with HIV-1 where there is no antiretroviral therapy (ART) [13–15]. Reasons for mixed feeding include social, cultural, tradition and individual factors of the mother [16–19]. Breastfeeding is traditional in Sub-Saharan Africa, but exclusive breastfeeding (EBF) is not [16, 19–24]. The prevalence of EBF ranges from 22 to 37% in this region, whereas it is estimated globally to be 35% [25, 26]. Factors known to negatively influence the practice of EBF are a) late initiation of breastfeeding (after one h postnatally), b) not giving colostrum, and c) provision of pre-lacteal feeds [25, 27, 28]. By giving ART to pregnant or breastfeeding women, post-natal HIV-transmission can be substantially reduced [29].

For these reasons, the World Health Organization (WHO) currently recommends HIV-infected pregnant women to breastfeed when formula is unsafe, i.e. as in most of Sub-Saharan Africa. At the time of the trial, exclusive breastfeeding for 6 months was recommended and thereafter, to introduce complementary food while continuing breastfeeding up to a year or until safe replacement feeding could be provided [30]. Potential strategies included the infant’s protection during the breastfeeding period, either by ART to the mother (option B or B+) or peri-exposure prophylaxis to the child (option A) [29–31]. However, maternal ART does not entirely eliminate postnatal MTCT, probably because of the persistence of a residual stable CD4+ T cell-associated reservoir of HIV-1 in breast milk [32]. Thus prolonged infant prophylaxis covering the entire recommended breastfeeding period was tested in the ANRS 12174 trial [33]. The trial aimed to compare the efficacy of lopinavir/ritonavir versus lamivudine to prevent the mother to child transmission of HIV-1 during breastfeeding period. The postnatal transmission rates were 1.4% (95% confidence interval [CI]; 0.4;2.5) and 1.5% (CI 0.7;2.5), respectively, in the lopinavir/ritonavir and lamivudine arms [34]. However, as breastfeeding is the main post-natal HIV exposure, and because its duration and modality (exclusive or not) are associated with postnatal transmission, we examined feeding practices and the determinants of exclusive and predominant breastfeeding practices of all participants in this trial.

## Methods

### Study design

The ANRS 12174 clinical trial in Ouagadougou (Burkina Faso), East London (South Africa), Mbale (Uganda) and Lusaka (Zambia) was conducted from 2009 to 2013. The protocol and the main outcome have been published [33, 34]. Briefly, HIV-1 infected pregnant women at the time ineligible for highly active antiretroviral therapy (HAART) because their CD4 count was >350 cells/mm3, aged 18 or above, planning to breastfeed and with between 28 and 40 weeks of amenorrhea, were identified from antenatal clinics. They received a pre-test counselling session before being tested for HIV infection. As part of the post-test session, they were informed of the different feeding options available for their babies. Only women wanting to breastfeed were referred to the research clinic for further assessment of the inclusion criteria during the antenatal period, and again with their child within 6 days after birth, for an enrolment and site-stratified randomisation at day 7 postpartum. From 28 weeks of pregnancy to day 7 after birth, programmatic mother to child transmission prophylaxis was implemented according to national guidelines, but mainly with antenatal zidovudine, an intrapartum single dose nevirapine and zidovudine-lamivudine for mothers and nevirapine for infants for 7 days postpartum. We excluded twins and triplets, infants positive in the HIV-1 DNA PCR test result at day 7 (±2 days) postpartum, low birth-weight or ill babies (ranked grade II or above of the DAIDS classification for adverse events) on the day of enrolment [35].

The intervention was infant prophylaxis in the breastfeeding period plus one week from day 7 to 50 weeks of age with either lopinavir/ritonavir or lamivudine. Lamivudine is generally well tolerated and accepted; it has been widely used in research and programs. Lopinavir/ritonavir paediatric formulation has been a promising prophylactic drug with low risks for resistance, high antiviral potency and a good safety profile [33]. However, it has poor palatability, which mattered less when introduced very early. Breastfeeding recalls of 24-h and one week were collected during the enrolment visit at day 7 ± 2 days after birth, and the 13 monthly scheduled follow-up visits starting at week 2. Prelacteal feeding data (which we defined as any food items except mothers’ milk given to infants before mothers initiated breastfeeding) were also collected at enrolment.

### Data management and analysis

Data were collected on case report forms or directly entered online using the Electronic Data capture system, viz. OpenClinica^®^ (https://www.openclinica.com). Based on the data collected at each visit, we categorized mothers into one of the following groups: 1) exclusive breastfeeding (EBF - only breast milk being given to the infant without any other kind of food or liquid except medically prescribed drugs or vitamins); 2) predominant breastfeeding (PBF - breast milk with some liquid-based food such as juice, tea, sugar-water and salt-water, including glucose without any kind of formula or animal milk); and 3) mixed feeding (MF - breast milk with other solid or other kind of milks with or without liquid-based food). We thereafter combined EBF and PBF into one group called “exclusive and predominant breastfeeding” (EPBF) as PBF presented few cases and was assessed to present the same risk than EBF at least with regard to postnatal HIV transmission [15]. The entire cohort was in this EPBF group at the beginning of the study and were followed up to detect any change to mixed feeding or non-breastfeeding by week 26 post-partum, the time when it is recommended to change from exclusive breastfeeding to complementary feeding. Any breastfeeding was defined as women breastfeeding whatever the pattern (exclusive, predominant or mixed feeding). Any breastfeeding applied during the whole duration of the study from day 7 to week 50 and was opposed to non-breastfeeding. We have also included women who stopped breastfeeding and eventually resumed it. We determined the number and mean duration of the periods with resumed breastfeeding between day 7 and week 50 postpartum.

For continuous variables, the median with inter-quartile range (IQR) were reported, and percentages for categorical variables. Comparison across categorical variables was done using the Chi-squared test. We described the participants’ feeding patterns using Kaplan-Meier survival curves for each country. The censoring date was the date of the child’s death, discontinuation, the last EPBF date or 22 weeks for those who completed the study without reporting any mixed feeding. The exit date for survival analysis was set at week 22, since prior to this time-point any stopping of EPBF was assumed to be participant-driven whereas at week 26 or later, it was induced by the trial requirements.

We also explored potentially modulating factors associated with early cessation of EPBF, as well as the early weaning before 50 weeks in a non-proportional hazard flexible parametric multiple regression analysis with duration of EPBF, or any breastfeeding as continuous variables using the “stpm2” command in Stata [36, 37]. Variables with a *p* ≤ 0.20 in the bivariate analysis were considered for the multivariate analysis in the pooled-data analysis of all the countries. Other covariates included the trial arm, trial country and the maternal age in 3 age groups (<25 years, 25 to 30 and 30+). The rest of the covariates were used as dichotomised variables (single vs. married or co-habiting, primi- vs. multipara, vaginal vs. caesarean section delivery, primary vs. secondary school or higher, breastfeeding initiation in time within the first hour postpartum vs. later. We also presented a country-specific analysis catering for contextual variation in factors associated with the feeding behaviour of the neonates. Since South Africa has a different socio-economic and cultural context, we stratified the data by creating 2 models - South Africa alone, and Uganda and Zambia together. Burkina Faso was excluded from these analyses due to the very small numbers of observations (women initiating mixed feeding in the study period before week 26). The same list of variables was used in the country-specific analysis regardless of the p-value in the bivariate analysis. Statistical analysis was done using STATA/SE 13.1 (4905 Lakeway Drive College Station, Texas 77845 USA).

### Ethics consent and permissions

Prior to enrolment, the mothers signed a written informed consent and assent form for themselves and their children, respectively. The trial was conducted according to the sponsor (ANRS) ethic charter, Good Clinical Practices and the principles of the Helsinki declaration. The protocol was approved by the relevant ethics committees in the four participating countries and the Medicines Control Council in South Africa.

## Results

### Baseline characteristics and feeding patterns

**Fig. 1:**
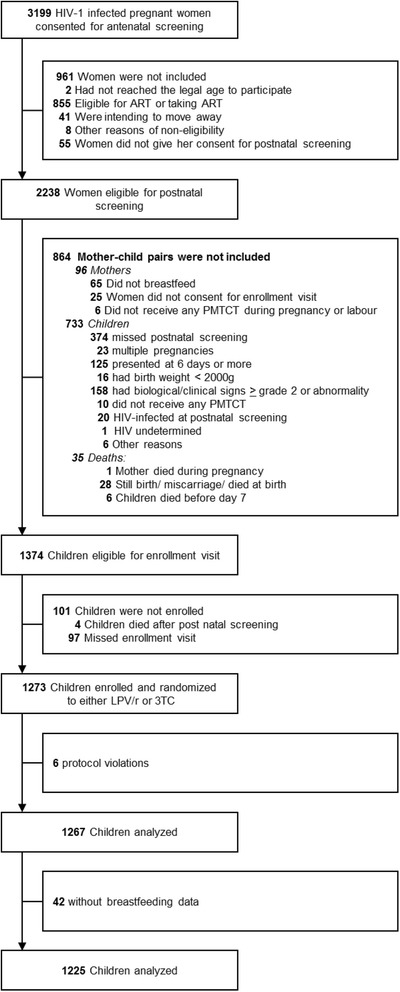
Study flow-chart

In the ANRS 12174 trial, 1,273 mother-infant pairs were randomized, of which 6 were excluded due to protocol violation. Of the remaining 1,267 participants, 204 were from Ouagadougou, 222 from East London, 278 from Mbale and 563 from Lusaka. Another 42 were excluded from analysis due to lack of breastfeeding data after inclusion (Fig. 1). Trial profile.

The mothers excluded from analysis tended to be younger (mean age 25 vs. 27 years), have better education and fewer children. Regarding baseline characteristics, South African mothers had the highest body mass index (BMI), and Ugandan mothers the highest number of children (Table 1).

**Table 1:** Study participants’ baseline characteristics

	Burkina Faso	South Africa	Uganda	Zambia	All sites
	*N* = 204	*N* = 213	*N* = 274	*N* = 534	*N* = 1225
	n (%)	n (%)	n (%)	n (%)	n (%)
Age group					
<25 years	54 (26.5)	73 (34.3)	107 (39.0)	200 (37.8)	434 (35.6)
25 – 30 years	75 (36.8)	67 (31.5)	97 (36.1)	175 (33.1)	414 (34.1)
30 and above	75 (36.8)	73 (34.3)	68 (24.8)	154 (29.0)	370 (30.3)
Literacy rate	103 (50.5)	211 (99.1)	261 (95.3)	487 (91.2)	1062 (86.7)
Education level (%)					
Primary school	155 (76.0)	19 (0.5)	176 (15.7)	249 (18.7)	599 (13.0)
Secondary school and higher	49 (24.0)	194 (91.1)	98 (35.7)	285 (53.4)	626 (51.1)
Occupation: employed	18 (8.8)	89 (41.8)	97 (35.4)	92 (17.2)	296 (24.2)
Married/co-habiting	185 (90.7)	83 (38.0)	225 (82.1)	473 (88.6)	966 (78.9)
Primiparous	44 (21.6)	71 (33.3)	49 (17.9)	112 (21.0)	276 (22.5)
Surgical breast history	8 (3.9)	1 (0.5)	8 (2.9)	1 (0.2)	18 (1.5)
Mother’s HIV stage 1	190 (93.1)	210 (98.6)	253 (92.3)	533 (99.8)	1184 (96.6)
Facility delivery	200 (98.0)	211 (99.1)	212 (77.4)	518 (97.0)	1141 (93.1)
Vaginal delivery	191 (93.6)	139 (65.3)	256 (93.4)	514 (96.2)	1100 (89.8)
Female infant	86 (42.2)	105 (49.3)	144 (52.5)	259 (48.5)	594 (48.5)
	Mean (95% CI)	Mean (95% CI)	Mean (95% CI)	Mean (95% CI)	Mean (95% CI)
Mean BMI	23.8 (23.2; 24.3)	28.3 (27.5; 29.0)	23.0 (22.6; 23.4)	24.1 (23.7; 24.4)	24.5 (24.3; 24.8)
Mean number of children	2.8 (2.6; 3.0)	2.0 (1.9; 2.2)	3.5 (3.3; 3.7)	2.6 (2.5; 2.7)	2.7 (2.7; 2.8)

Breastfeeding was initiated within 1 h and on the first day postpartum by 57.7% and 93.9% of mothers, respectively (Table 2), and 99% had started any breastfeeding within the first week (Table 3). The main reason for delayed initiation of breastfeeding was reported as a lack of breast milk. EBF, PBF and MF were practiced by 95.9, 1.6 and 1.5%, respectively, in the first week (Table 3). Water-based liquids were the most common prelacteal items (6.2% of the participants) during this week (Table 4).

**Table 2:** Time to initiation of breastfeeding after birth

Hours	Burkina Faso	South Africa	Uganda	Zambia	All sites
	*N* = 204	*N* = 213	*N* = 274	*N* = 534	*N* = 1225
	n (%)	n (%)	n (%)	n (%)	n (%)
0-1	14 (6.9)	109 (51.2)	152 (55.5)	432 (80.9)	707 (57.7)
2-5	73 (35.8)	85 (39.9)	76 (27.7)	74 (13.9)	308 (25.1)
6-12	54 (26.5)	10 (4.7)	32 (11.7)	22 (4.1)	118 (9.6)
12-24	4 (2.0)	3 (1.4)	6 (2.2)	4 (0.7)	17 (1.4)
Total initiated 1st day	145 (71.1)	207 (97.2)	266 (97.1)	532 (99.6)	1150 (93.9)

**Table 3:** Feeding pattern during the first week of life by country

Breast feeding pattern	Burkina Faso	South Africa	Uganda	Zambia	All sites
	*N* = 204	*N* = 213	*N* = 274	*N* = 534	*N* = 1225
	n (%)	n (%)	n (%)	n (%)	n (%)
Any breastfeeding first 3 days	200 (98.0)	206 (96.7)	272 (99.3)	532 (99.6)	1210 (98.8)
Any breastfeeding day 4-7	202 (99.0)	205(96.2)	273 (99.6)	533 (99.8)	1213 (99.0)
Exclusive breastfeeding throughout first 3 days	156 (76.5)	199 (93.4)	250 (91.2)	528 (98.9)	1133 (92.5)
Exclusive breastfeeding throughout days 4-7	177 (86.8)	200 (93.9)	266 (97.1)	532 (99.6)	1175 (95.9)
Predominant breastfeeding throughout first 3 days	34 (16.7)	2 (0.9)	18 (6.6)	1 (0.2)	55 (4.5)
Predominant breastfeeding throughout days 4-7	15 (7.3)	4 (1.9)	1 (0.4)	0 (0.0)	20 (1.6)
Mixed feeding first 3 days	10 (4.9)	5 (2.3)	4 (1.5)	3 (0.6)	22 (1.8)
Mixed feeding day 4-7	10 (4.9)	1 (0.5)	6 (2.2)	1 (0.2)	18 (1.5)

**Table 4:** Proportion of infants receiving different food items during the first week of life by country

Nutrient or other intake	Burkina Faso	South Africa	Uganda	Zambia	All sites
	*N* = 204	*N* = 213	*N* = 274	*N* = 534	*N* = 1225
	n (%)	n (%)	n (%)	n (%)	n (%)
Days 1 to 3					
Breast milk	200 (98.0)	206 (96.7)	272 (99.3)	532 (99.6)	1210 (98.8)
Water	34 (16.7)	5 (2.3)	12 (4.4)	0 (0.0)	51 (4.2)
Water + sugar or glucose	6 (2.9)	0 (0.0)	15 (5.5)	0 (0.0)	21 (1.7)
Water + salt	0 (0.0)	0 (0.0)	4 (1.5)	0 (0.0)	4 (0.3)
Juice	1 (0.5)	0 (0.0)	0 (0.0)	1 (0.2)	2 (0.2)
Cow’s milk	0 (0.0)	0 (0.0)	0 (0.0)	1 (0.2)	1 (0.1)
Infant formula	2 (1.0)	4 (1.9)	0 (0.0)	3 (0.6)	9 (0.7)
Liquid as part of traditional practice	6 (2.9)	1 (0.5)	3 (1.1)	0 (0.0)	10 (0.8)
Other	2 (1.0)	0 (0.0)	2 (0.4)	0 (0.0)	3 (0.2)
Days 4 to 7					
Breast milk	20 2(99.0)	205 (96.2)	273 (99.6)	533 (99.8)	1213 (99.0)
Water	19 (9.3)	4 (1.9)	1 (0.4)	0(0.0)	24 (2.0)
Water + sugar or glucose	1 (0.5)	1 (0.5)	1 (0.4)	0 (0.0)	3 (0.2)
Tea	1 (0.5)	0 (0.0)	0 (0.0)	0 (0.0)	1 (0.1)
Juice	3 (1.5)	0 (0.0)	0 (0.0)	0 (0.0)	3 (0.2)
Infant formula	1 (0.5)	0 (0.0)	1 (0.4)	1 (0.2)	3 (0.2)
Powdered milk	1 (0.5)	0 (0.0)	0 (0.0)	0 (0.0)	1 (0.1)
Liquid as part of traditional practice	6 (2.9)	1 (0.5)	3 (1.1)	0 (0.0)	10 (0.8)
Other	3 (1.5)	0 (0.0)	1 (0.4)	0 (0.0)	4 (0.3)

The median duration of any breastfeeding was 40.6 weeks (interquartile range (IQR; 32.3; 45.4; Table 5). Burkina Faso had the longest median duration and South Africa the shortest. The median duration of EBF was 20.9 (IQR: 19.1; 21.1) weeks. PBF was reported only during 59 monthly visits (4/1000 person-year). When PBF was combined with EBF, the overall median duration remained the same (20.9; IQR: 19.7; 21.1).

**Table 5:** Median duration of “any breastfeeding”, “exclusive breastfeeding” and “exclusive and predominant breastfeeding” in weeks

Feeding patterns	Burkina Faso	South Africa	Uganda	Zambia	All sites
	*N* = 204	*N* = 213	*N* = 274	*N* = 534	*N* = 1225
	Median (p25; p75)	Median (p25; p75)	Median (p25; p75)	Median (p25; p75)	Median (p25; p75)
Any breastfeeding	46.5 (45.0; 48.7)	29.1 (13.0; 46.3)	39.9 (34.1; 43.0)	39.0 (33.0; 43.6)	40.6 (32.3; 45.4)
Exclusive breastfeeding	20.7 (18.9; 21.3)	19.5 (12.4; 21.0)	20.9 (19.6; 21.0)	21.0 (20.4; 21.1)	20.9 (19.1; 21.1)
Exclusive and predominant breastfeeding	20.9 (20.0; 21.5)	19.8 (12.9; 21.0)	20.9 (19.9; 21.0)	21.0 (20.6; 21.1)	20.9 (19.7; 21.1)

**Fig. 2:**
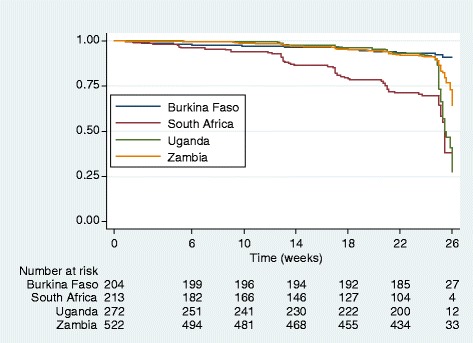
Kaplan-Meier survival curve by country for exclusive and predominant breastfeeding until week 26 post-partum

At week 22, EPBF was practiced by 90.7, 48.8, 79.6 and 83.1% in Burkina Faso, South Africa, Uganda and Zambia, respectively (Fig. 2 and Additional file 1). Nevertheless at week 22 there was no significant difference between the trial arms (*p* = 0.05, log rank test; in the lopinavir/ritonavir arm, 70.3% practiced EBF vs. 69.1% in the lamivudine arm).

**Fig. 3:**
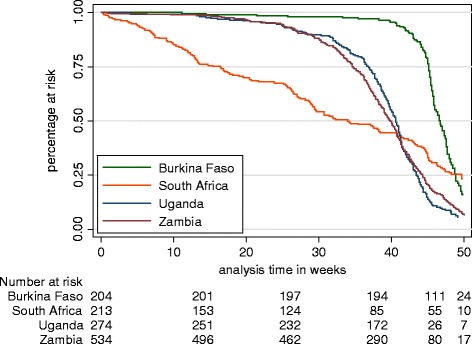
Kaplan-Meier survival curve by country for any breastfeeding until week 50 post-partum

At week 50, 11.8, 4.7, 2.5 and 3.2% of the mothers were continuing to breastfeed in Burkina Faso, South Africa, Uganda and Zambia, respectively (Fig. 3).

The details of the food items that the infants received are set out in detail in Table 4 (first week) and in the Additional files 2, 3 (first year). There were major country differences in prelacteal feeds (Table 4). In the first 3 days of life, water-based items (including water, water and sugar or glucose, water and salt, cow’s milk, infant formula and traditional beverages) were given to 24, 12.5, 4.7 and 1% of the children in Burkina Faso, Uganda, South Africa and Zambia, respectively. After the first week of life (Additional file 2, 3), milk items were mainly used in South Africa, Uganda and Zambia. Porridge or cereals were were introduced at week 10 and 18 in Zambia and Uganda, respectively while in Burkina Faso they were used from week 26. Soup, meat, fish or egg were introduced as early as week 18 in South Africa, Uganda and Zambia while these items were first seen in infant’s food only at week 26 (0.5% for meat, fish or egg) in Burkina Faso (additional file 2c). After cessation of breastfeeding, 62 (5.1%) of 1225 mothers (25 (11.7%), 20 (7.3%) and 17 (3.2%) in South Africa, Zambia and Uganda, respectively) resumed breastfeeding. In total, 54 (4.4%) mothers resumed breastfeeding once and stopped breastfeeding permanently, and 8 (0.6%) resumed it twice. The mean duration of all periods with resumed breastfeeding was 17.2 days, and in calculating this mean, we disregarded 5 women who had resumed breastfeeding of long duration (mean duration of 108.2 days) and were characterized as outliers. The mean duration of these periods with resumed breastfeeding for mothers who resumed once was 18.2 days (i.e. without these 5 women), 14.9 and 13.2 days for the first and second period, respectively, for those who resumed twice. We did not find any difference between the women who stopped breastfeeding earlier (before 26 weeks) and resumed it and those who stopped it later (after 26 weeks).

### Risk factors analysis

Almost all the Burkina Faso mothers continued EPBF beyond 22 weeks after birth. We therefore removed them from the pooled multivariable analysis on risk factors for stopping EPBF (Additional file 2: Table S6). The groups of mothers significantly more at risk to stop EPBF before 26 weeks in the remaining 3 countries were the 25-30 year-old married, employed, and multiparous mothers, or those in the lopinavir/ritonavir arm. On the other hand, Ugandan and Zambian women were more likely to continue EPBF until 26 weeks than South African women. The behaviour of South African women to stop EPBF early affected the overall analysis, showing that the lopinavir/ritonavir arm was a risk factor in this situation.

With regard to any breastfeeding, Burkina Faso was once again dropped from the multivariate analysis of the determinants. The survival curve (Fig. 3) of any breastfeeding by country shows a dramatic drop of the South African curve from the beginning until around week 40. From week 40, the remaining South African mothers tended to breastfeed longer than those mothers in the other countries. Having completed secondary school or beyond was an independent risk factor for early cessation of any breastfeeding (Additional file 2: Table S6).

In the country-specific analyses (Additional file 2: Table S7a), there were in general no large differences in the infant feeding behavior, except for South Africa where there was an interaction with trial allocation. Children in the lopinavir/ritonavir group were 3 times more likely to stop EPBF early than children from the lamivudine group. In Uganda and Zambia, the trial drug had no significant influence on infant feeding behavior.

Contrary to any breastfeeding, having a secondary school or higher education level was beneficial for EPBF practice, allowing up to 40, 60 and 40 of babies to benefit from EPBF in South Africa, Uganda and Zambia, respectively. However, this association was significant only for Zambian children. Being married or living as a couple was a risk factor for shorter EPBF, with a significant adjusted hazard ratio (AHR) of 1.6 (95% CI: 1.2; 2.1) and 2.6 (95% CI: 1.9; 3.6) in South Africa and Zambia, respectively. A similar pattern was observed with employed mothers and multiparous mothers. Late initiation of breastfeeding (after 1 h) was associated with shorter EPBF in Uganda and Zambia.

## Discussion

In general, the mothers in this trial adhered to the breastfeeding recommendations: 58% initiated breastfeeding within the first hour and 94% within the first day. EPBF was practiced at a high rate in all countries throughout the 22-week period. This performance can probably be attributed to the study design, including close tracking of the mothers during the antenatal period and frequent follow-up visits with infant feeding counselling. The median durations of any and exclusive breastfeeding were 40.6 and 20.9 weeks, respectively. At week 22, >75% of the children were on EPBF. Only 4.7% were breastfed at week 50.

In other recent studies of both the general population and mothers living with HIV, findings showed that the proportion of women initiating breastfeeding within the first hour postpartum was between less than half and 76% [18, 38–40]. However, with respect to our study, the country having the lowest percentage of women initiating breastfeeding within the first hour was Burkina Faso (only 6.9%).

Under 10% of the participants in this trial gave prelacteal feeds compared to 22 to 57% in other studies [18, 20, 39, 40]. Burkina Faso had the highest proportion of women giving prelacteal feeds (>16%). The same feeding patterns were found in the PROMISE-EBF study in Burkina Faso site with <4 and 11% of participants initiating breastfeeding within the first hour and giving prelacteal feeding, respectively [41]. In Burkina Faso, however, women breastfed longer compared to the other countries, for which we have no clear explanation. Our hypotheses are that the tradition through a cultural influence, better counselling sessions and a poorer economic context that could be accompanied with difficulties in affording quality complementary food for children, may have been important factors. More mothers in South Africa (and to some extent in Zambia) had to resume formal work after a few months, preventing them from continuing breastfeeding.

In the Kesho Bora study [42], the participants from Burkina Faso had a considerably lower level of education, than in our study. In contrast to our study, South Africa had the longest median duration of any breastfeeding compared with Burkina Faso and Kenya. The proportion of women initiating breastfeeding in the first week and the median duration of any breastfeeding were higher in our study (99% and 41 weeks vs. 70% and 20 weeks, respectively). Likewise, the Kesho Bora study had a lower proportion of EBF (22% at 5 months) compared to our study (75.3% at 5 months). Even comparing the countries that participated in both the Kesho Bora and our trial (Burkina Faso and South Africa), the exclusive breastfeeding rate was higher in our study. In other EBF studies with contexts similar to this trial, the median durations of EBF varied from 1.8 [43] to 5 months [38, 43, 44] and the proportion of EBF at 6 months ranged from zero percent to 84% [18, 20, 42, 43, 45, 46]. Therefore one has to acknowledge the high variability of infant feeding practices in sub-Saharan Africa among HIV-positive women. In general, the ANRS12174 participants adhered to a large extent to the WHO HIV and infant feeding recommendations. The trial setup and the frequent counselling is the most likely explanation for that; thus, it is possible that frequent quality counselling is required for the promotion of the safe infant feeding practices among HIV-1 positive women.

Interestingly, the trial allocation was a factor associated with breastfeeding behaviour, but only in South Africa where the lopinavir/ritonavir group had a 3 times higher risk of shorter EPBF compared to the lamivudine group. It is possible that the poor palatability of lopinavir/ritonavir influenced the South African participants to stop EPBF earlier than recommended. Why this happened only in South Africa is unclear, but formula-feeding is more common in South Africa, which may be one explanation. It is also possible that, due to the bad taste, women in South Africa mixed the drug with other foods than milk, unlike women in Uganda and Zambia where they mixed it with breastmilk.

Our findings together with the ANRS 12174 trial results [34] demonstrated enough that breastfeeding in HIV-1 infected women could be almost as safer as in HIV-1 uninfected mothers for the infants, provided it is practised according to the international recommendations. However, it is currently commonly accepted to breastfeed babies born to HIV-1 infected mothers while the peri-exposure prophylaxis is assured by the mothers taking lifelong anti-retroviral medicines (option B+). Nonetheless, the efficacy demonstrated for the option A in our studies required some reflections on how to combine all these options for an improved PMTCT strategy as we know that some HIV reservoirs (including in breastmilk) [32] are not fully controlled by the mother’s HAART. In the other hands, some mothers may not be able to afford or to take HAART.

There are certain limitations to our study. In some sites, including Burkina Faso, the nutritional counsellors were also collecting feeding data; therefore, there was a risk for the mothers to under-report non-recommended practices while emphasizing the recommended ones. However, the counsellors were senior staff with considerable experience in research and knew the techniques needed to probe participants to obtain accurate data. Furthermore, the Burkina Faso participants had the highest proportion of prelacteal feeding, delayed initiation of breastfeeding, among other items, which suggests that information bias was limited. Moreover, our findings regarding prelacteal feeding practices and breastfeeding initiation time in Burkina Faso are comparable with PROMISE-EBF findings [41], where peer counsellors were not the same as the investigators who collected the data.

Other potential limitations are the trial context with a fixed time of 50 weeks for provision of the infant prophylaxis, which limits the generalisability of our data particularly on ‘any breastfeeding’, and the selection criteria that may not allow any extrapolation of the results to non-research situations. However, our large sample size, the long duration of our follow-up, the international multicentre design, as well as the stringent randomised clinical trial context and the cohort design, added value and accuracy to our findings.

## Conclusion

ANRS 12174 trial participants were relatively more successful in practicing EPBF than has been seen in several previous studies. However, in Burkina Faso, late initiation of breastfeeding postpartum and the extensive use of prelacteal feeds remain prevalent. Why women in the lopinavir/ritonavir arm were more likely to stop EPBF in South Africa is a question requiring further investigation. There is a need to improve breastfeeding and complementary feeding practices of children, particularly those exposed to HIV and anti-retrovirals, taking into account context and socio-demographic factors.

## Additional files

Additional file 1: Figure S4. Non-exclusive breastfeeding survival curves by country until week 50. This figure shows survival curves by country presenting the women nonexclusively breastfeeding their children during the 50-week follow-up period. (PDF 115 kb)

Additional file 2: Table S6. Non-proportional hazard models of early cessation of ‘exclusive or predominant breastfeeding’ and ‘any breastfeeding’ [As a result table]. This table presents overall factors determining early cessation of exclusive or predominant and any breastfeeding. **Table S7a**. Flexible parametric non-proportional hazard models of shorter duration of ‘exclusive or predominant breastfeeding’ by country. [As a result table]. This table gives factors determining early cessation of exclusive or predominant breastfeeding stratified by country. **Table S7b**. Flexible parametric non-proportional hazard models of shorter duration of ‘exclusive or predominant breastfeeding’ isolating South-Africa. [As a result table]. This table details factors determining early cessation of exclusive or predominant and any breastfeeding stratified by country in 2 different models, including South African model alone and another model for Uganda and Zambia together. (DOCX 47.1 kb)

Additional file 3: Table S8a. Infant feeding practices in detail: liquid-based items given during the study period. This additional file is a table describing the different liquid-based food items other than breastmilk given to the child during the study follow-up period. **Table S8b**. Infant feeding practices in detail: milk-based items given during the study period. This additional file is a table describing the different milk-based food items other than breastmilk given to the child during the follow-up period. **Table S8c**. Infant feeding practices in detail: solids items given during the study period. This additional file is a table describing the different solid food items other than breastmilk given to the child during the study follow-up period. (DOCX 52.9 kb)

## Abbreviations

ART: Antiretroviral therapy
BMI: Body mass index
EBF: Exclusive breastfeeding
EPBF: Exclusive or predominant breastfeeding
HAART: Highly active antiretroviral therapy
HIV: Human Immuno-deficiency Virus
IQR: Inter-quartile range
MF: Mixed feeding
MTCT: Mother-to-child transmission of HIV-1
PBF: Predominant breastfeeding
PMTCT: Prevention of mother-to-child transmission of HIV-1
UNAIDS: United Nations programme on HIV/AIDS
WHO: World Health Organization
