# Supplementary figures and images for: Breastfeeding patterns and its determinants among mothers living with Human Immuno-deficiency Virus -1 in four African countries participating in the ANRS 12174 trial
Source: Int Breastfeed J. 2017 May 2;12:22. doi: 10.1186/s13006-017-0112-2 (PMC5414228; doi:10.1186/s13006-017-0112-2)

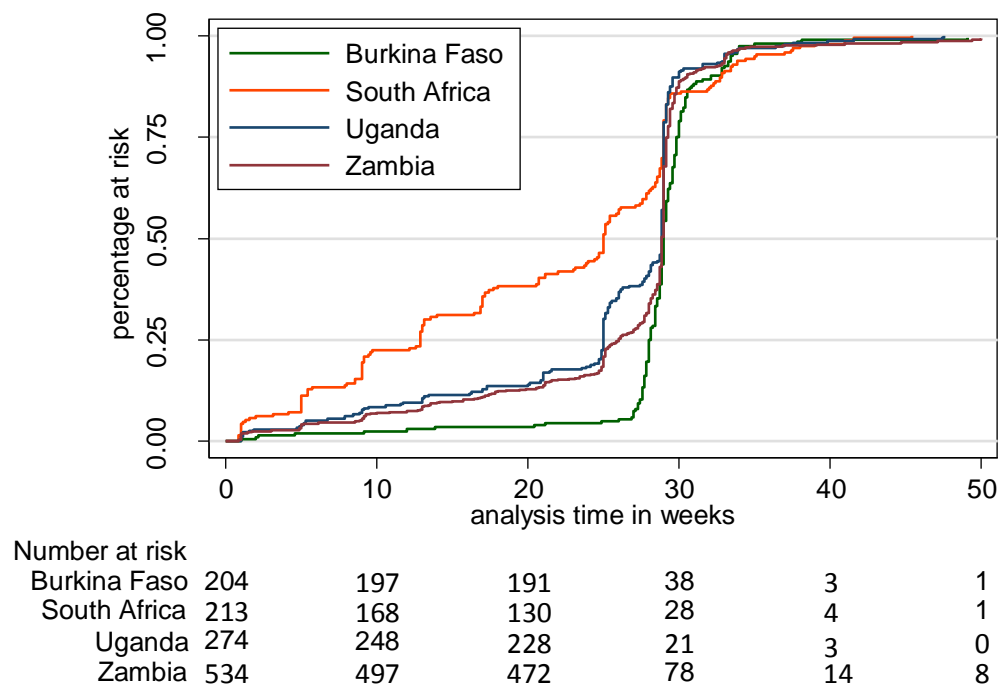

Additional file 1 : Non exclusive breastfeeding survival curves by country until week 50

Supplement: Supplementary file 1 — Non-exclusive breastfeeding survival curves by country until week 50. This figure shows survival curves by country presenting the women nonexclusively breastfeeding their children during the 50-week follow-up period. (PDF 115 kb) [file 13006_2017_112_MOESM1_ESM.pdf]
