# Supplementary material for: Breastfeeding patterns and its determinants among mothers living with Human Immuno-deficiency Virus -1 in four African countries participating in the ANRS 12174 trial
Source: Int Breastfeed J. 2017 May 2;12:22. doi: 10.1186/s13006-017-0112-2 (PMC5414228; doi:10.1186/s13006-017-0112-2)
Supplement: Supplementary file 2 — Non-proportional hazard models of early cessation of ‘exclusive or predominant breastfeeding’ and ‘any breastfeeding’ [As a result table]. This table presents overall factors determining early cessation of exclusive or predominant and any breastfeeding. Table S7a. Flexible parametric non-proportional hazard models of shorter duration of ‘exclusive or predominant breastfeeding’ by country. [As a result table]. This table gives factors determining early cessation of exclusive or predominant breastfeeding stratified by country. Table S7b. Flexible parametric non-proportional hazard models of shorter duration of ‘exclusive or predominant breastfeeding’ isolating South-Africa. [As a result table]. This table details factors determining early cessation of exclusive or predominant and any breastfeeding stratified by country in 2 different models, including South African model alone and another model for Uganda and Zambia together. (DOCX 47.1 kb) [file 13006_2017_112_MOESM2_ESM.docx]

Additional files

Table S6: Non-proportional hazard models of early cessation of ‘exclusive or predominant breastfeeding’ and ‘any breastfeeding’.

|  | **Early cessation of ‘exclusive or predominant breastfeeding’** | |  | **Early cessation of ‘any breastfeeding’** | |
| --- | --- | --- | --- | --- | --- |
|  | **Crude HR^a^**  **(95% CI^b^)** | **Adjusted HR^a^**  **(95% CI^b^)** |  | **Crude HR^a^**  **(95% CI^b^)** | **Adjusted HR^a^**  **(95% CI^b^)** |
| Trial arm |  |  |  |  |  |
| Lamivudine | 1 | 1 |  | 1 | 1 |
| Lopinavir/Ritonavir | 1.3 (1.0; 1.5) | 1.4 (1.2; 1.7) |  | 1.0 (0.9; 1.1) | 1.3 (1.2; 1.4) |
| Country |  |  |  |  |  |
| South Africa | 1 | 1 |  | 1 | 1 |
| Uganda | 0.2 (0.1; 0.3) | 0.2 (0.1; 0.3) |  | 1.0 (0.9; 1.1) | 1.4 (1.3; 1.5) |
| Zambia | 0.2 (0.1; 0.3) | 0.3 (0.2; 0.4) |  | 1.0 (0.9; 1.1) | 1.3 (1.2; 1.5) |
| Mothers’ age |  |  |  |  |  |
| Below 25 years | 1 | 1 |  | 1 | 1 |
| Between 25 – 30 | 1.5 (1.2; 1.8) | 1.5 (1.2; 1.8) |  | 1.1 (1.0; 1.2) | 1.2 (1.1; 1.3) |
| 30 and above | 1.2 (1.0; 1.4) | 1.0 (0.8; 1.2) |  | 0.9 (0.8; 1.0) | 1.0 (0.9; 1.1) |
| Education |  |  |  |  |  |
| Completed primary | 1 | 1 |  | 1 | 1 |
| Secondary and more | 1.9 (1.6; 2.2) | 0.8 (0.7; 1.0) |  | 1.2 (1.1; 1.3) | 1.2 (1.1; 1.3) |
| Marital |  |  |  |  |  |
| Single | 1 | 1 |  | 1 | 1 |
| Married | 2.9 (2.5; 3.4) | 1.8 (1.5; 2.2) |  | 1.0 (0.9; 1.1) | 1.0 (0.9; 1.1) |
| Occupation |  |  |  |  |  |
| Not employed | 1 | 1 |  | 1 | 1 |
| Employed | 1.6 (1.3; 1.8) | 1.4 ( 1.1;1.6) |  | 1.2 (1.1; 1.3) | 1.2 (1.1; 1.3) |
| Parity: |  |  |  |  |  |
| Primiparous | 1 | 1 |  | 1 | 1 |
| Multiparous | 0.9 (0.8; 1.2) | 1.4 (1.1; 1.8) |  | 0.9 (0.8; 1.0) | 0.9 (0.9; 1.0) |
| Delivery mode |  |  |  |  |  |
| Vaginal | 1 | 1 |  | 1 | 1 |
| C-section | 1.8 (1.4; 2.2) | 0.8 (0.6; 1.0) |  | 1.1 (1.0; 1.2) | 1.2 (1.1; 1.3) |
| Breastfeeding initiation |  |  |  |  |  |
| Within 1^st^ hour | 1 | 1 |  | 1 | 1 |
| After 1^st^ hour | 1.4 (1.2; 1.7) | 1.1 (0.9; 1.3) |  | 0.9 (0.8; 1.0) | 0.9 (0.8; 1.0) |

^a^ Hazard Ratio ^b^ 95% confidence interval

Table S7a: Flexible parametric non-proportional hazard models of shorter duration of ‘exclusive or predominant breastfeeding’ by country.

|  | **South Africa** |  |  | **Uganda** |  |  | **Zambia** |  |
| --- | --- | --- | --- | --- | --- | --- | --- | --- |
|  | **Crude HR^a^**  **(95% CI^b^)** | **Adjusted HR^a^**  **(95% CI^b^)** |  | **Crude HR^a^**  **(95% CI^b^)** | **Adjusted HR^a^**  **(95% CI^b^)** |  | **Crude HR^a^**  **(95% CI^b^)** | **Adjusted HR^a^**  **(95% CI^b^)** |
| Trial arm |  |  |  |  |  |  |  |  |
| Lamivudine | 1 | 1 |  | 1 | 1 |  | 1 | 1 |
| Lopinavir/ritonavir | 2.7 (1.6; 4.5) | 3.0 (1.6; 5.5) |  | 0.5 (0.2; 1.4) | 0.4 (0.1; 1.3) |  | 0.9 (0.7; 1.2) | 1.0 (0.7; 1.3) |
| Maternal age group |  |  |  |  |  |  |  |  |
| Below 25 years | 1 | 1 |  | 1 | 1 |  | 1 | 1 |
| Between 25 – 30 | 1.6 (1.2; 2.1) | 1.4 (1.1; 1.9) |  | 1.7 (1.1; 2.7) | 1.5 (0.8; 2.5) |  | 1.3 (1.0; 1.8) | 1.3 (1.0; 1.9) |
| 30 and above | 0.8 (0.6; 1.1) | 0.7 (0.5; 1.0) |  | 0.9 (0.5; 1.6) | 0.9 (0.5; 1.7) |  | 1.5 (1.1; 2.1) | 1.3 (0.9; 1.9) |
| Education level |  |  |  |  |  |  |  |  |
| Completed primary | 1 |  |  | 1 | 1 |  | 1 | 1 |
| Secondary & higher | 0.9 (0.6; 1.5) | 0.6 (0.3; 1.1) |  | 1.5 (1.0; 2.3) | 0.4 (0.1; 1.3) |  | 0.9 (0.7; 1.2) | 0.6 (0.4; 0.8) |
| Marital status |  |  |  |  |  |  |  |  |
| Single | 1 | 1 |  | 1 | 1 |  | 1 | 1 |
| Married/cohabiting | 1.4 (1.1; 1.8) | 1.6 (1.2; 2.1) |  | 1.1 (0.7; 1.9) | 1.1 (.7; 1.9) |  | 2.2 (1.6; 2.0) | 2.6 (1.9; 3.6) |
| Occupation |  |  |  |  |  |  |  |  |
| Not employed | 1 | 1 |  | 1 | 1 |  | 1 | 1 |
| Employed | 1.2 (0.9; 1.5) | 1.3 (1.0; 1.6) |  | 1.5 (1.0; 2.3) | 1.3 (0.9; 2.0) |  | 1.4 (1.0; 1.9) | 1.5 (1.1; 2.1) |
| Parity |  |  |  |  |  |  |  |  |
| Primiparous | 1 | 1 |  | 1 | 1 |  | 1 | 1 |
| Multiparous | 1.3 (1.0; 1.7) | 1.6 (1.2; 2.1) |  | 1.0 (0.6; 1.7) | 1.2 (0.6; 2.2) |  | 1.3 (0.9; 1.9) | 1.5 (1.0; 2.2) |
| Mode of delivery |  |  |  |  |  |  |  |  |
| Vaginal | 1 | 1 |  | - | - |  | 1 | 1 |
| C-section | 0.8 (0.6; 1.0) | 0.9 (0.7; 1.2) |  | - | - |  | 1.3 (0.7; 2.3) | 1.0 (0.6; 1.9) |
| Breastfeeding initiation |  |  |  |  |  |  |  |  |
| Within 1^st^ hour | 1 | 1 |  | 1 | 1 |  | 1 | 1 |
| After 1^st^ hour | 0.8 (0.6 ; 1.0) | 0.9 (0.7 ; 1.1) |  | 2.0 (1.3 ; 3.1) | 1.8 (1.2; 2.9) |  | 1.3 (0.9 ; 1.7) | 1.2 (0.9 ; 1.6) |

^a^ Hazard Ratio; ^b^ 95% confidence interval

Table S7b: Flexible parametric non-proportional hazard models of shorter duration of ‘exclusive or predominant breastfeeding’ isolating South-Africa.

|  | | **Exclusive or predominant breastfeeding** | | | | **Any breastfeeding** | | | |
| --- | --- | --- | --- | --- | --- | --- | --- | --- | --- |
|  | | **South Africa** |  | **Uganda and Zambia** | | **South Africa** |  | **Uganda and Zambia** | |
|  | **Crude HR^a^**  **(95% CI^b^)** | | **Adjusted HR^a^**  **(95% CI^b^)** | **Crude HR^a^**  **(95% CI^b^)** | **Adjusted HR^a^**  **(95% CI^b^)** | **Crude HR^a^**  **(95% CI^b^)** | **Adjusted HR^a^**  **(95% CI^b^)** | **Crude HR^a^**  **(95% CI^b^)** | **Adjusted HR^a^**  **(95% CI^b^)** |
| Trial arm |  | |  |  |  |  |  |  |  |

| Lamivudine | 1 | 1 | 1 | 1 | 1 | 1 | 1 | 1 |
| --- | --- | --- | --- | --- | --- | --- | --- | --- |
| Lopinavir/ritonavir | 2.7 (1.6; 4.5) | 3.0 (1.6; 5.5) | 0.9 (0.7; 1.2) | 0.9 (0.7; 1.2) | 2.0 (1.6; 2.4) | 2.2 (1.8; 2.7) | 1.2 (1.1; 1.4) | 1.2 (1.1; 1.4) |
| Maternal age group |  |  |  |  |  |  |  |  |
| Below 25 years | 1 | 1 | 1 | 1 | 1 | 1 | 1 | 1 |
| Between 25 – 30 | 1.6 (1.2; 2.1) | 1.4 (1.1; 1.9) | 1.4 (1.1; 1.9) | 1.4 (1.0; 1.8) | 1.1 (0.9; 1.3) | 1.0 (0.9; 1.2) | 1.0 (0.9; 1.1) | 1.1 (1.0; 1.2) |
| 30 and above | 0.8 (0.6; 1.1) | 0.7 (0.5; 1.0) | 1.4 (1.0; 1.8) | 1.3 (0.9; 1.7) | 0.6 (0.5; 0.7) | 0.6 (0.5; 0.7) | 1.0 (0.9; 1.1) | 1.1 (0.9; 1.2) |
| Education level |  |  |  |  |  |  |  |  |
| Completed primary | 1 |  | - | - | 1 | 1 | 1 | 1 |
| Secondary & higher | 0.9 (0.6; 1.5) | 0.6 (0.3; 1.1) | - | - | 1.5 (1.2; 2.0) | 1.1 (0.8; 1.6) | 1.3 (1.2; 1.3) | 1.2 (1.1; 1.4) |
| Marital status |  |  |  |  |  |  |  |  |
| Single | 1 | 1 | 1 | 1 | - | - | - | - |
| Married/cohabiting | 1.4 (1.1; 1.8) | 1.6 (1.2; 2.1) | 1.7 (1.3; 2.3) | 1.9 (1.4; 2.5) | - | - | - | - |
| Occupation |  |  |  |  |  |  |  |  |
| Not employed | 1 | 1 | 1 | 1 | 1 | 1 | 1 | 1 |
| Employed | 1.2 (0.9; 1.5) | 1.3 (1.0; 1.6) | 1.4 (1.1; 1.7) | 1.3 (1.0; 1.6) | 1.3 (1.1; 1.5) | 1.3 (1.1; 1.5) | 1.2 (1.1; 1.3) | 1.2 (1.1; 1.3) |
| Parity |  |  |  |  |  |  |  |  |
| Primiparous | 1 | 1 | 1 | 1 | 1 | 1 | 1 | 1 |
| Multiparous | 1.3 (1.0; 1.7) | 1.6 (1.2; 2.1) | 1.2 (0.9; 1.6) | 1.2 (0.9; 1.7) | 0.8 (0.7; 0.9) | 1.0 (0.8; 1.2) | 0.9 (0.9; 1.0) | 0.9 (0.8; 1.0) |
| Mode of delivery |  |  |  |  |  |  |  |  |
| Vaginal | 1 | 1 | - | - | - | - | 1 | 1 |
| C-section | 0.8 (0.6; 1.0) | 0.9 (0.7; 1.2) | - | - | - | - | 1.3 (1.2; 1.5) | 1.5 (0.3; 1.7) |
| Breastfeeding initiation |  |  |  |  |  |  |  |  |
| Within 1^st^ hour | 1 | 1 | 1 | 1 | 1 | 1 | 1 | 1 |
| After 1^st^ hour | 0.8 (0.6; 1.0) | 0.9 (0.7 ; 1.1) | 1.4 (1.1; 1.7) | 1.3 (1.0; 1.6) | 1.2 (1.0; 1.3) | 1.2 (1.0; 1.4) | 0.9 (0.8; 1.0) | 0.8 (0.7; 0.9) |

^a^ Hazard Ratio; ^b^ 95% confidence interval
