# Supplementary material for: Breastfeeding patterns and its determinants among mothers living with Human Immuno-deficiency Virus -1 in four African countries participating in the ANRS 12174 trial
Source: Int Breastfeed J. 2017 May 2;12:22. doi: 10.1186/s13006-017-0112-2 (PMC5414228; doi:10.1186/s13006-017-0112-2)
Supplement: Supplementary file 3 — Infant feeding practices in detail: liquid-based items given during the study period. This additional file is a table describing the different liquid-based food items other than breastmilk given to the child during the study follow-up period. Table S8b. Infant feeding practices in detail: milk-based items given during the study period. This additional file is a table describing the different milk-based food items other than breastmilk given to the child during the follow-up period. Table S8c. Infant feeding practices in detail: solids items given during the study period. This additional file is a table describing the different solid food items other than breastmilk given to the child during the study follow-up period. (DOCX 52.9 kb) [file 13006_2017_112_MOESM3_ESM.docx]

Additional file 3

Table S8a: Infant feeding practices in detail: liquid-based items given during the study period.

| **Country** | **W2**  **n (%)** | **W6**  **n (%)** | **W10**  **n (%)** | **W14**  **n (%)** | **W18**  **n (%)** | **W22**  **n (%)** | **W26**  **n (%)** | **W30**  **n (%)** | **W34**  **n (%)** | **W38**  **n (%)** | **W42**  **n (%)** | **W46**  **n (%)** | **W50**  **n (%)** | **Total**  **n (%)** |
| --- | --- | --- | --- | --- | --- | --- | --- | --- | --- | --- | --- | --- | --- | --- |
| **Water** | | | | | | | | | | | | | | |
| BF | 10(4.9) | 3(1.5) | 0 (0) | 2 (1.0) | 1 (0.5) | 5 (2.4) | 12 (5.9) | 190(93.1) | 190(93.1) | 186(91.2) | 187(91.7) | 181(88.7) | 194(95.1) | 1161(43.8) |
| SA | 2 (1.0) | 1(0.5) | 3(1.6) | 8 (3.9) | 11(5.3) | 10(4.8) | 16 (7.8) | 58 (28.2) | 63(30.6) | 67(32.5) | 58(28.2) | 52(25.2) | 44(21.4) | 393(14.7) |
| Uganda | 0 (0) | 0 (0) | 0 (0) | 1 (0.4) | 0 (0) | 1 (0.4) | 20 (7.3) | 114(41.9) | 163(59.9) | 151(55.5) | 93(34.2) | 18(6.6) | 12(4.4) | 573(16.2) |
| Zambia | 1 (0.2) | 1(0.2) | 2(0.9) | 4 (0.7) | 5 (0.9) | 18(3.4) | 57(10.7) | 428(80.3) | 442(82.9) | 445(83.5) | 433(81.2) | 418(78.4) | 457(85.7) | 2711(39.1) |
| **Water-sugar or glucose** | | | | | | | | | | | | | | |
| BF | 0 | 0 | 0 | 0 | 0 | 0 | 1 (0.5) | 3(1.5) | 10(4.9) | 9(4.4) | 20(9.8) | 22(10.8) | 27(13.2) | 92(3.6) |
| SA | 0 | 0 | 0 | 0 | 0 | 0 | 0 | 1(0.5) | 1(0.5) | 0 | 0 | 0 | 1(0.5) | 3(0.1) |
| Uganda | 0 | 1(0.4) | 0 | 0 | 0 | 0 | 0 | 3(1.1) | 2(0.7) | 0 | 0 | 0 | 0 | 6(0.2) |
| Zambia | 0 | 0 | 0 | 1 (0.2) | 1 (0.2) | 2 (0.4) | 10 (1.9) | 56(10.5) | 63(11.8) | 46(8.6) | 46(8.6) | 40(7.5) | 31(5.8) | 296(4.3) |
| **Water with salt** | | | | | | | | | | | | | | |
| BF | 0 | 0 | 0 | 0 | 1(0.5) | 0 | 2(1.0) | 0 | 2(1.0) | 0 | 0 | 3(1.5) | 1(0.5) | 9(0.3) |
| SA | 0 | 0 | 0 | 0 | 0 | 0 | 0 | 1(0.5) | 0 | 0 | 0 | 0 | 0 | 1(0.04) |
| Uganda | 0 | 1(0.4) | 0 | 0 | 0 | 0 | 0 | 0 | 0 | 0 | 0 | 0 | 0 | 1(0.03 |
| Zambia | 0 | 0 | 0 | 0 | 1(0.2) | 1(0.2) | 3(0.6) | 20(3.7) | 17(3.2) | 14(2.6) | 19(3.6) | 9(1.7) | 9(1.7) | 93(1.3) |
| **Tea** | | | | | | | | | | | | | | |
| BF | 0 | 0 | 0 | 0 | 0 | 0 | 0 | 1(0.5) | 4(2.0) | 1(0.5) | 1(0.5) | 4(2.0) | 2(1.0) | 13(0.5) |
| SA | 0 | 0 | 0 | 0 | 2(1.0) | 5(2.4) | 9(4.4) | 52(25.2) | 58(28.2) | 56(27.2) | 48(23.3) | 46(22.3) | 39(18.9) | 315(11.8) |
| Uganda | 0 | 0 | 0 | 0 | 0 | 0 | 7(2.6) | 58(21.3) | 81(29.8) | 78(28.7) | 60(22.1) | 11(4.0) | 6(2.2) | 301(8.5) |
| Zambia | 0 | 0 | 0 | 0 | 1(0.2) | 4(0.7) | 7(1.3) | 138(25.9) | 254(47.6) | 329(61.7) | 371(69.6) | 373(70.0) | 425(79.7) | 1902(27.4) |
| **Juice** | | | | | | | | | | | | | | |
| BF | 0 | 0 | 1(0.5) | 0 | 0 | 2(1.0) | 1(0.5) | 73(35.8) | 72(35.3) | 70(34.3) | 68(33.3) | 75(36.8) | 79(38.7) | 441(16.6) |
| SA | 0 | 0 | 0 | 0 | 2(1.0) | 5(2.4) | 10(4.8) | 61(29.6) | 60(29.1) | 61(29.6) | 53(25.7) | 47(22.8) | 40(19.4) | 339(12.7) |
| Uganda | 0 | 0 | 0 | 0 | 0 | 0 | 3(1.1) | 11(4.0) | 24(8.8) | 21(7.7) | 15(5.5) | 2(0.7) | 3(1.1) | 79(2.2) |
| Zambia | 0 | 1(0.2) | 0 | 1(0.2) | 2(0.4) | 4(0.7) | 22(4.1) | 292(54.8) | 363(68.1) | 407(76.4) | 405(76.0) | 402(75.4) | 444(83.3) | 2343(33.8) |
| **Honey** | | | | | | | | | | | | | | |
| BF | 0 | 0 | 0 | 0 | 0 | 0 | 1(0.5) | 1(0.5) | 3(1.5) | 2(1.0) | 0 | 1(0.5) | 1(0.5) | 9(0.3) |
| SA | 0 | 0 | 0 | 0 | 0 | 0 | 0 | 0 | 0 | 0 | 0 | 0 | 0 | 0 |
| Uganda | 0 | 0 | 0 | 0 | 0 | 0 | 1(0.4) | 0 | 1(0.4) | 1(0.4) | 1(0.4) | 0 | 0 | 4(0.1) |
| Zambia | 0 | 0 | 0 | 0 | 0 | 0 | 0 | 0 | 0 | 0 | 1(0.2) | 1(0.2) | 2(0.4) | 4(0.06) |
| **Traditional treatment: enema** | | | | | | | | | | | | | | |
| BF | 4(2.0) | 1(0.5) | 0 | 0 | 0 | 1(0.5) | 0 | 6(2.9) | 7(3.4) | 5(2.4) | 5(2.4) | 8(3.9) | 8(3.9) | 45(1.7) |
| SA | 0 | 0 | 1(0.5) | 0 | 0 | 0 | 0 | 0 | 0 | 0 | 0 | 0 | 0 | 1(0.04) |
| Uganda | 0 | 0 | 0 | 0 | 0 | 0 | 0 | 0 | 1(0.4) | 0 | 0 | 0 | 0 | 1(0.03 |
| Zambia | 0 | 0 | 0 | 0 | 0 | 0 | 0 | 0 | 0 | 0 | 0 | 0 | 0 | 0 |
| **Traditional treatment: herbs** | | | | | | | | | | | | | | |
| BF | 6(2.94) | 0 | 0 | 0 | 0 | 0 | 0 | 4(2.0) | 2(1.0) | 5(2.4) | 2(1.0) | 1(0.5) | 1(0.5) | 21(0.8) |
| SA | 0 | 0 | 1(0.5) | 0 | 0 | 0 | 0 | 0 | 0 | 0 | 0 | 0 | 0 | 1(0.04) |
| Uganda | 0 | 0 | 0 | 0 | 0 | 0 | 0 | 0 | 0 | 0 | 0 | 0 | 0 | 0 |
| Zambia | 0 | 0 | 0 | 0 | 1(0.2) | 1(0.2) | 0 | 0 | 0 | 0 | 0 | 0 | 0 | 2(0.03) |
| **Alcohol** | | | | | | | | | | | | | | |
| BF | 0 | 0 | 0 | 0 | 0 | 1(0.5) | 1(0.5) | 1(0.5) | 4(2.0) | 3(1.5) | 6(2.9) | 4(2.0) | 6(2.9) | 25(0.9) |
| SA | 0 | 0 | 0 | 0 | 0 | 0 | 0 | 0 | 0 | 0 | 0 | 1(0.5) | 0 | 1(0.04) |
| Uganda | 0 | 0 | 0 | 0 | 0 | 0 | 0 | 1(0.4) | 1(0.4) | 0 | 0 | 0 | 0 | 2(0.06) |
| Zambia | 0 | 0 | 0 | 1(0.2) | 0 | 0 | 0 | 0 | 0 | 0 | 0 | 0 | 0 | 1(0.01) |

Table S8b: Infant feeding practices in detail: milk-based items given during the study period.

| **Country** | **W2**  **n (%)** | **W6**  **n (%)** | **W10**  **n (%)** | **W14**  **n (%)** | **W18**  **n (%)** | **W22**  **n (%)** | **W26**  **n (%)** | **W30**  **n (%)** | **W34**  **n (%)** | **W38**  **n (%)** | **W42**  **n (%)** | **W46**  **n (%)** | **W50**  **n (%)** | **Total**  **n (%)** |
| --- | --- | --- | --- | --- | --- | --- | --- | --- | --- | --- | --- | --- | --- | --- |
| **Diluted milk** | | | | | | | | | | | | | | |
| BF | 0 | 0 | 0 | 0 | 1 (0.5) | 0 | 0 | 0 | 1 (0.5) | 0 | 0 | 0 | 2 (1.0) | 4 (0.02) |
| SA | 0 | 0 | 0 | 0 | 2 (1.0) | 5 (2.4) | 10 (4.8) | 58 (28.2) | 64 (31.1) | 68 (33.0) | 57 (27.7) | 51 (24.8 | 43 (20.9) | 358 (13.4) |
| Uganda | 0 | 0 | 0 | 2 (0.7) | 2 (0.7) | 3 (1.1) | 38 (13.9) | 112 (41.2) | 115 (42.3) | 88 (32.35  ) | 52 (19.1) | 7 (2.6) | 8 (2.9) | 427 (12.1) |
| Zambia | 0 | 0 | 0 | 0 | 0 | 2 (0.4) | 11 (2.1) | 72 (13.5) | 104 (19.5) | 119 (22.3 ) | 118 (22.1) | 131 (24.6) | 146 (27.4) | 703 (10.1) |
| **Undiluted milk** | | | | | | | | | | | | | | |
| BF | 0 | 0 | 0 | 0 | 0 | 0 | 0 | 2 (1.0) | 0 | 2 (1.0) | 0 | 3 | 1 | 0 |
| SA | 0 | 0 | 0 | 0 | 3 (1.5) | 4 (1.9) | 12 (5.8) | 59 (28.6) | 65 (31.5) | 68 (33.0) | 58 (28.2) | 48 (23.3) | 42 (20.4) | 359 (13.4) |
| Uganda | 0 | 0 | 0 | 0 | 0 | 0 | 6 (2.2) | 10 (3.7) | 6 (2.2) | 7 (2.6) | 3 (1.1) | 0 | 0 | 32 (0.9) |
| Zambia | 1(0.2) | 0 | 0 | 2(0.4) | 1(0.2) | 2(0.4) | 2(0.4) | 30(5.6) | 44(8.3) | 64(12.0) | 80(15.0) | 93(17.4) | 99(18.6) | 418(6.0) |
| **Infant formula** | | | | | | | | | | | | | | |
| BF | 2 | 2 | 1 | 1 | 1 | 1 | 2 (1.0) | 6 | 4 | 4 | 4 | 18 | 36 | 82 |
| SA | 4 (2.0) | 7 (3.4) | 5 (2.43) | 17 (8.2) | 17 (8.2) | 20 (9.7) | 20 (9.7) | 14 (6.8) | 14 (6.8) | 16 (7.8) | 23 (11.2) | 16 (7.8) | 10 (4.8) | 183 (6.8) |
| Uganda | 0 | 0 | 0 | 0 | 0 | 0 | 0 | 1 (0.37) | 1 (0.37) | 0 | 0 | 0 | 0 | 2 (0.06) |
| Zambia | 0 | 0 | 2 (0.4) | 2 (0.4) | 4 (0.7) | 7 (1.3) | 10 (1.9) | 27 (5.1) | 17 (3.2) | 17 (3.2) | 20 (3.7) | 18 (3.4) | 28 (5.2) | 152 (2.2) |
| **Powdered milk** | | | | | | | | | | | | | | |
| BF | 0 | 0 | 0 | 0 | 0 | 0 | 0 | 1 | 0 | 3 | 8 | 18 | 35 | 65 |
| SA | 0 | 0 | 0 | 1 (0.5) | 2 (1.0) | 4 (2.0) | 11 (5.3) | 56 (27.2) | 69 (33.5) | 71 (34.5) | 56 (27.2) | 54 (26.2) | 43 (20.9) | 367 (13.7) |
| Uganda | 0 | 0 | 0 | 0 | 1 (0.4) | 0 | 0 | 1 (0.4) | 2 (0.7) | 0 | 0 | 0 | 0 | 4 (0.1) |
| Zambia | 0 | 0 | 0 | 0 | 0 | 0 | 1 (0.2) | 4 (0.7) | 4 (0.7) | 7 (1.3) | 2 (0.4) | 3 (0.6) | 5 (0.9) | 26 (0.4) |
| **Other non-maternal milk/dairy product** | | | | | | | | | | | | | | |
| BF | 0 | 0 | 0 | 0 | 0 | 0 | 0 | 8 | 20 | 35 | 36 | 51 | 73 | 223 |
| SA | 0 | 0 | 1 (0.4) | 0 | 1 (0.5) | 4 (1.9) | 11 (5.3) | 57 (27.7) | 70 (34.0) | 73 (35.4) | 57 (27.7) | 54 (26.2) | 44 (21.4) | 372 (13.9) |
| Uganda | 0 | 0 | 0 | 0 | 0 | 0 | 0 | 0 | 0 | 2 (0.74) | 0 | 0 | 0 | 2 (0.1) |
| Zambia | 0 | 1 (0.2) | 0 | 0 | 2 (0.4) | 3 (0.6) | 20 (3.7) | 198 (37.1) | 259 (48.6) | 284 (53.3) | 274 (51.41) | 278 (52.2) | 332 (62.3) | 1651(23.8) |

Table S8c: Infant feeding practices in detail: solid items given during the study period.

|  | **W2**  **n (%)** | **W6**  **n (%)** | **W10**  **n (%)** | **W14**  **n (%)** | **W18**  **n (%)** | **W22**  **n (%)** | **W26**  **n (%)** | **W30**  **n (%)** | **W34**  **n (%)** | **W38**  **n (%)** | **W42**  **n (%)** | **W46**  **n (%)** | **W50**  **n (%)** | **Total**  **n (%)** |
| --- | --- | --- | --- | --- | --- | --- | --- | --- | --- | --- | --- | --- | --- | --- |
| **Porridge or cereals** | | | | | | | | | | | | | | |
| BF | 0 | 0 | 0 | 0 | 0 | 0 | 2 (0.98) | 167 (81.86) | 169 (82.84) | 172 (84.31) | 173 (84.80) | 174 (85.29) | 193 (94.61) | 1050 (39.59) |
| SA | 1 (0.5) | 0 (0.0) | 0 (0.0) | 2(1.0) | 6(2.9) | 7(3.4) | 15(7.3) | 69(33.5) | 77(37.4) | 73(35.4) | 62(30.1) | 54(26.2) | 44(21.4) | 410(15.3) |
| Uganda | 0 | 0 | 0 | 0 | 1(0.4) | 2(0.7) | 28(10.3) | 142(52.2) | 145(53.3) | 118(16.5) | 81(7.0) | 18(1.1) | 11(0.4) | 543(49.1) |
| Zambia | 0 | 0 | 2 (0.4) | 4(0.7) | 6(1.1) | 17(3.2) | 61(11.4) | 427(80.1) | 441(82.7) | 447(83.9) | 433(81.2) | 417(78.2) | 456(85.5) | 2711(39.1) |
| **Soup** | | | | | | | | | | | | | | |
| BF | 0 | 0 | 0 | 0 | 0 | 0 | 0 | 48(23.5) | 81(39.7) | 69(33.8) | 64(31.4) | 73(35.8) | 79(38.7) | 414(15.6) |
| SA | 0 | 0 | 0 | 0 | 3(1.5) | 6(2.9) | 14(6.8) | 66(32.0) | 77(37.4) | 72(34.9) | 62(30.1) | 54(26.2) | 44(21.4) | 398(14.9) |
| Uganda | 0 | 0 | 0 | 0 | 0 | 0 | 10(3.7) | 114(41.9) | 152(55.9) | 132(48.5) | 81(29.8) | 16(5.9) | 10(3.7) | 515(14.6) |
| Zambia | 0 | 0 | 0 | 0 | 2(0.4) | 5(0.9) | 25(4.7) | 382(71.7) | 428(80.3) | 440(82.5) | 429(80.5) | 419(78.6) | 455(85.4) |  |
| **Meat, fish or egg** | | | | | | | | | | | | | | |
| BF | 0 | 0 | 0 | 0 | 0 | 0 | 1(0.5) | 20(9.8) | 45(22.1) | 61(29.9) | 78(38.2) | 89(43.6) | 114(55.9) | 408(15.4) |
| SA | 0 | 0 | 0 | 0 | 4(1.9) | 5(2.4) | 15(7.3) | 68(33.0) | 77(37.4) | 72(34.9) | 62(30.1) | 54(26.2) | 44(21.4) | 401 (15.0) |
| Uganda | 0 | 0 | 0 | 0 | 0 | 0 | 3(1.1) | 39(14.3) | 66(24.3) | 71(26.1) | 46(16.9) | 9(3.3) | 5(1.8) | 239(6.76) |
| Zambia | 0 | 0 | 0 | 0 | 2(0.4) | 2(0.4) | 21(3.9) | 335(62.8) | 406(76.2) | 424(79.5) | 426(79.9) | 414(77.7) | 452(84.8) | 2482(35.8) |
| **Other** | | | | | | | | | | | | | | |
| BF | 0 | 0 | 0 | 0 | 0 | 0 | 0 | 37(18.1) | 59(28.9) | 77(37.7) | 85(41.7) | 91(44.6) | 113(55.4) | 462(17.4) |
| SA | 0 | 2(1.0) | 1(0.5) | 0 | 0 | 0 | 1(0.5) | 0 | 0 | 1(0.5) | 0 | 0 | 0 | 5(0.2) |
| Uganda | 0 | 0 | 0 | 0 | 0 | 1(0.4) | 7(2.6) | 123(45.2) | 158(58.1) | 152(55.9) | 91(33.5) | 17(6.2) | 11(4.0) | 560(15.8) |
| Zambia | 1(0.2) | 0 | 0 | 1(0.2) | 1(0.2) | 0 | 0 | 0 | 1(0.2) | 0 | 0 | 0 | 2(0.4) | 6(0.1) |
